# Trends in Prevalence of Yeast Species Associated With Urogenital Infection in Nsukka, Nigeria: An Overview of True *Candida* Species and Genotyping of *Candida albicans hwp1*-Heterozygous Isolates

**DOI:** 10.1155/ijm/3115363

**Published:** 2025-09-17

**Authors:** Eunice Ngozi Anaele, Josephine I. Okafor, Lucilla Lo Re, Carla Lo Passo, Francesco Mediati, Grazia Galeano, Orazio Romeo, Roberta Galbo, Letterio Giuffrè

**Affiliations:** ^1^South-East Zonal Biotechnology Centre, University of Nigeria, Nsukka, Enugu State, Nigeria; ^2^Department of Microbiology, University of Nigeria, Nsukka, Enugu State, Nigeria; ^3^Department of Chemical, Biological, Pharmaceutical and Environmental Sciences, University of Messina, Messina, Italy; ^4^Functional Gastrointestinal Disorders Research Group, National Institute of Gastroenterology IRCCS “Saverio de Bellis”, Castellana Grotte, Italy

**Keywords:** *Candida albicans*, genotyping, multilocus sequence typing, urogenital infection

## Abstract

Pathogenic drug-resistant yeast species, associated with urogenital infections, are still not well-recognized in routine clinical laboratories. This study describes the prevalence and antifungal susceptibility profile of fungal species isolated from patients with urogenital infection in Nsukka, Nigeria. A total of 248 urogenital samples (voided urine, high vaginal swabs, urethral swabs, and semen) were cultured on specific mycological media for the isolation and presumptive identification of *Candida* and other yeast species. Further identification of fungal isolates was performed using conventional phenotypic techniques and molecular methods. Disk diffusion and broth dilution methods were used for the antifungal susceptibility study and determination of minimum inhibitory concentration (MIC), respectively. A total of 129 *yeasts* were isolated from 117 patients with urogenital infection. *Candida albicans* (73.64%) was the most prevalent species followed by *Pichia kudriavzevii* (*Candida krusei*) (9.30%) and *Candida parapsilosis* (6.20%), while *Nakaseomyces glabrata* (*Candida glabrata*) and *Candida tropicalis* exhibited the same frequency of occurrence (5.43% each). All *Candida* isolates were susceptible to voriconazole and nystatin, while reduced susceptibility to fluconazole was noted. All the germ tube–positive isolates were confirmed to be *C. albicans* by molecular methods although 15 of them were found to be heterozygous at *hwp*1 locus. This study describes the distribution of true *Candida* species causing urogenital infection in Nigeria and the level of susceptibility of these species to common antifungal drugs emphasizing the need for yeast culture and antifungal susceptibility testing as part of the routine test in medical diagnostic laboratories for the proper management of urogenital candidiasis.

## 1. Introduction

Urogenital candidiasis is an infection of the urinary and genital tracts caused by pathogenic *Candida* species which may manifest as vaginitis in women, balanitis and balanoposthitis in men, and candiduria in both sexes [1]. This disease is not strictly regarded as sexually transmitted, but women can pass *Candida* to their partners during vaginal intercourse [2]. Urogenital infection can affect the bladder, kidney, vagina, cervix, urethra, periurethral, ureters, penis, prostate, epididymis, and testicles, affecting fertility in both males and females [3–5].

In men, urogenital candidiasis leads to inflammation of the glans penis and foreskin, itchy rash on the genital, discharge from penis, and painful urination, while in women, vulvovaginal candidiasis (VVC) is the second most common vaginitis after bacterial vaginosis. Infection in women is characterized by pelvic and vulvar pain, itching, painful urination, and severe pain during sex. The source of the etiologic agent of VVC is endogenous, due to the overgrowth of the normal fungal flora in the presence of a predisposing condition [6] which usually includes the use of antibiotics, corticosteroids, oral contraceptives, immunosuppression, diabetes, and poor hygiene. It has been estimated that 75% of women of reproductive age suffer from VVC at least once during their lifetime, while 8% complain of recurrent infection (three or more episodes of VVC within a year) [7, 8]. Urogenital infection can also manifest as candiduria, a common nosocomial fungal infection among hospitalized patients due to the extensive use of broad-spectrum antimicrobials, corticosteroids or immunosuppressive drugs, diabetes mellitus, and in-dwelling catheters [9].

The increasing incidence of urogenital candidiasis and the emergence of *Candida* isolates resistant to antifungal drugs have been recognized as a health challenge with serious implications both in the developed and developing countries [10, 11]. The current situation calls for proper management of urogenital candidiasis through regular surveillance of the distribution of *Candida* species and susceptibility of the isolates to the antifungals [12, 13]. Recent reports have shown that variation in *Candida* species distribution was dependent on geographic location, socioeconomic status, *ethnicity*, and underlying medical conditions [14, 15]. However, several studies have endorsed *Candida albicans* as the dominant species in urogenital infection [15–17], while recent reports have shown the dramatic emergence of various nonalbicans *Candida* (NAC) species, such as *Candida glabrata* and *Candida krusei*, with intrinsic resistance to fluconazole, including the new globally emerging pathogen *Candida auris* [12, 13, 18]. However, it should be noted that several studies still consider *C. glabrata* and *C. krusei* as NAC species, although these two yeasts are taxonomically no longer considered members of the *Candida* genus. In fact, *C. krusei* is currently known as *Pichia kudriavzevii*, while *C. glabrata* has been reclassified as *Nakaseomyces glabrata* [19]. Therefore, continuing to include these two species in the NAC group could potentially influence the global epidemiology of candidiasis, leading to an incorrect estimate of the true prevalence of *Candida* spp., with a direct impact on pharmacotherapy, public health, and decision-making.

In Nigeria, the few existing reports on the distribution of pathogenic fungi and the antifungal susceptibility profiles of the isolates from different clinical samples indicate that the epidemiology of these pathogens varies from one region to another [3, 20], but there is a paucity of data on the distribution of *Candida* spp. in urogenital samples from male and female patients in southeastern Nigeria. Therefore, the aim of the present study was to determine the incidence, distribution, and antifungal susceptibility of pathogenic yeast species recovered from patients with complaint of urogenital infection in Nsukka, Enugu State, Nigeria.

## 2. Materials and Methods

### 2.1. Sample Collection and Culturing

Based on the patient's consent and with the approval of healthcare management, urogenital samples, from patients who self-reported symptoms of genitourinary infection and patients with the doctors' recommendation for culture of urine or genital samples in healthcare centers, were recruited for the study. Such healthcare centers include Bishop Shanahan Hospital, University of Nigeria Medical Center, All Saints Medical Center, and Kenol Medical Diagnostic Laboratory in Nsukka, Enugu State, Nigeria. This study included patients aged 20–45 years who were sexually active and self-reported symptoms of genitourinary infection or individuals with a doctor's recommendation to have urine culture or genital samples taken at health centers. Patients who were on antifungal drug, diabetic, pregnant, and HIV positive were excluded from the present study. For isolation of *Candida* spp. and other pathogenic yeast, mid-stream urine, high vaginal swabs (HVSs), urethral swabs (USs) and semen samples were collected and cultured on Sabouraud dextrose agar (SDA) with chloramphenicol (50 mg/L). The inoculated SDA plates were incubated at 37°C for 72 h under aerobic condition. A total of 248 urogenital samples were recovered in this study.

### 2.2. Phenotypic Identification of Fungal Species

The isolated yeast colonies were subcultured on chromogenic CHROMagar *Candida* medium for presumptive species identification and recognition of mixed cultures. Standard mycological phenotypic methods of identification were also employed and included sugar assimilation tests using the API ID32C system (*bioMérieux*, France), germ tube formation in serum at 37°C for 2 h, and chlamydospore production on corn meal agar at 25°C for 5 days [20].

### 2.3. Molecular Identification of True *Candida* Species and Other Pathogenic Yeasts

Species-specific PCR-based methods were employed for definitive identification of *C. albicans*, *Candida tropicalis*, *Candida parapsilosis*, *N. glabrata* (formerly *C. glabrata*), and their phylogenetically closely related species/biovariants [21]. The identity of *P. kudriavzevii* isolates (formerly *C. krusei*) was confirmed by direct sequencing of the purified PCR products obtained by amplifying the two i*nternal transcribed* spacer *regions* (*ITS1* and *ITS2*), including the 5.8S rRNA region, with the universal fungal primers ITS1 and ITS4 [22].

Genomic DNA was isolated as described by Müller et al. using glass bead disruption of the fungal cells followed by a traditional DNA extraction protocol based on phenol–chloroform purification and ethanol precipitation [23]. The pellet was suspended in 30 *μ*L of TE buffer and stored at −20°C until use [23].

All *Candida* isolates that produced green-colored colonies on CHROMagar *Candida*, germ tubes, and/or chlamydospores were subjected to molecular identification based on partial amplification of the hyphal wall Protein 1 (*hwp1*) gene as previously described by Romeo and Criseo [24]. Other species, whose colony color was typical of that of *C. tropicalis* (dark blue) or *N. glabrata* (*C. glabrata*) (mauve), were identified using the PCR amplification of the vacuolar membrane ATPase (*VMA*) *intein gene* [25] and the multiplex PCR protocol reported by Romeo et al. [26], respectively. *C. parapsilosis* isolates were identified by using the multiplex PCR assay as described by Asadzadeh et al. [27].

All PCR amplifications were carried out in a MyCycler thermal cycler (Bio-Rad, Italy) using the DreamTaq PCR master mix (Thermo Fisher Scientific, Italy) plus 1 *μ*L of genomic DNA template and 0.5 *μ*M of each primer, depending on the PCR assay used [24–27]. PCR conditions were set according to each specific molecular method employed.

PCR products were separated on a 1.3% (wt/vol) agarose gel, stained with ethidium bromide, and visualized/analyzed under ultraviolet light using a transilluminator (Moonlight, Diatech, Italy). The sizes of amplified DNA fragments were determined by comparison with a 100-bp DNA ladder (GeneRuler, Thermo Fisher, Italy) and positive controls (standard strains used: *C. albicans* ATCC 10231, *C. glabrata* ATCC 90030, *C. tropicalis* CBS13074, and *C. parapsilosis* ATCC 22019).

### 2.4. Multilocus Sequence Typing (MLST) of *hwp1*-Heterozygous *C. albicans* Isolates

All *C. albicans* isolates, showing a heterozygous genotype at *hwp1* locus, were subjected to MLST analysis following the genotyping scheme based on partial amplification and sequencing of seven (*AAT1a*, *ACC1*, *ADP1*, *MPIb*, *SYA1*, *VPS13*, and *ZWF1b*) housekeeping protein-coding genes [28, 29]. The PCR mixture, primers, and cycling parameters used for each MLST marker were the same as those reported in our previous study [30]. Purified PCR amplicons were Sanger-sequenced, in both directions, at the Eurofins Genomics, Ebersberg, Germany (http://www.eurofinsgenomics.eu), using the same MLST-PCR oligonucleotide primers.

Each forward and reverse sequencing electropherogram was visually inspected with the FinchTV V1.4 software (Geospiza Inc., Seattle, WA) to detect calling errors and identify potential heterozygous sites. Next, for each fungal isolate, DNA sequences from the seven MLST loci were used to query the international *C. albicans* MLST database (https://pubmlst.org/organisms/candida-albicans) to assign allele numbers and define the diploid sequence type (DST) [29, 30]. New allelic combinations (new DSTs) were submitted to the official *C. albicans* MLST database, and accession numbers were provided by the curator.

Phylogenetic and population structure analysis of *C. albicans* was performed as described by Scordino et al. [30]. The dataset used for this analysis included all MLST data of the 15 Nigerian isolates along with 3553 unique DSTs and 5548 isolates available in the official *C. albicans* PubMLST database (last accessed 11/04/2024). Dendrograms were constructed using the unweighted pair group method with an arithmetic mean (UPGMA) algorithm in MEGA Ver. 11 (http://www.megasoftware.net) with p-distance model and 1000 bootstrap replicates, while clonal complexes and putative founder genotypes were predicted using the goeBURST algorithm implemented in the PHYLOViZ 2.0 software (http://www.phyloviz.net).

### 2.5. Antifungal Susceptibility Testing of Yeast Isolates

The disk diffusion method, recommended by Clinical and Laboratory Standards Institute (CLSI), was used [31]. The Mueller–Hinton agar plate supplemented with 2% glucose and 0.5 *μ*g/mL methylene blue was inoculated with sterile swab dipped in standardized *Candida* inoculum (0.5 McFarland standard) to obtain a uniform growth to cover the entire plate. Antifungal drug disks (Oxoid, United Kingdom), purchased from Oxoid distributor, Vic Domstell Company in Lagos, Nigeria, were used. Fluconazole (25 *μ*g), voriconazole (1 *μ*g), and nystatin (100 units) disks were placed on the surface of the inoculated agar. The plates were incubated at 37°C for 48 h. The inhibition zone diameter (IZD) was measured to the nearest millimeter using a transparent ruler. The assay was performed in duplicate.

### 2.6. Determination of the Fluconazole Minimum Inhibitory Concentration (MIC)

The CLSI broth macrodilution reference method M27-A3 [32] was used with little modification. Fluconazole pure drug powder was obtained as a kind gift from Juhel Pharmaceutical Company, Awka, Anambra State, Nigeria. The drug (25.6 mg powder) was dissolved in sterile distilled water (100 mL) and twofold serially diluted to get a final drug concentration ranging from 256 to 4 *μ*g/mL. In another set of test tubes, 0.1 mL of standardized *Candida* inoculum was added to 0.4 mL sterile Sabouraud dextrose broth giving a total of 0.5 mL per test tube. Then, 0.5 mL of each twofold drug concentration was mixed with 0.5 mL of the broth containing standardized *Candida* inoculum in a test tube. The test tubes were incubated at 37°C for 2 days (48 h). The control was made up of 0.5 mL of the broth containing the standardized *Candida* inoculum supplemented with 0.5 mL sterile distilled water. The resultant MIC was the lowest concentration of fluconazole that showed a prominent decrease in turbidity when compared with the growth control. The MIC value was the lowest concentration of the drug in the broth medium that inhibited a 50% visible growth of the test microbe in comparison with the control. The MIC results were interpreted using species-specific CLSI breakpoints as recommended by the M27M44S document [33]. *C. albicans*, *C. parapsilosis*, and *C. tropicalis* strains with MIC of ≥ 8 *μ*g/mL were considered resistant to fluconazole, while, for *N. glabrata* (*C. glabrata*), the resistant MIC cutoff value was ≥ 64 *μ*g/mL [33].

### 2.7. Statistical Analysis

The data obtained were presented as means and percentages and analyzed using the chi-square test. The differences in means were adjudged to be significant at *p* < 0.05. Statistical Package for Social Sciences (SPSS 23.0) was used.

## 3. Results

### 3.1. Prevalence and Species Distribution of Yeast Isolates

Of 248 patients with symptoms of urogenital infection, yeasts were isolated from 117 (~47.18%) (Table 1). The prevalence of candiduria was 44.85% (61/136), while genital candidiasis was 50% (56/112) (Table 1). No significant difference was observed in the occurrence of *Candida* in urine and genitals of patients with symptoms of urogenital infection (*p* > 0.05). Three *Candida* species (*C*. *albicans*, *C*. *parapsilosis*, and *C*. *tropicalis*) as well as *P. kudriavzevii* (*C. krusei*) and *N. glabrata* (*C. glabrata*) [19] were identified from urine and HVS samples, while only *C*. *albicans* was isolated and identified from US (Table 1). None of the semen samples yielded growth of *Candida* species or other pathogenic yeasts. The most dominant yeast species was *C*. *albicans* accounting for 73.64%, while *C*. *parapsilosis* (6.20%) was the most common NAC species followed by both *C. tropicalis* and *N. glabrata* (*C. glabrata*) (5.43% each) (Table 1). *P. kudriavzevii* was more frequent than individual NAC species (9.3%) (Table 1). However, if we exclude *P. kudriavzevii* and *N. glabrata* from the epidemiological analysis and consider only the true *Candida* species (110 strains in total) (Table 1), the incidence of *C. albicans* raise to ~86.4%, while that of *C. parapsilosis* and *C. tropicalis* increases by approximately one percentage point.

*C*. *albicans* was the only yeast species isolated from males (urine, 20.59%; US, 25%) (Table 2), while, in females, all three species of *Candida*, including *P. kudriavzevii* and *N. glabrata*, were recovered (Table 2). The occurrence of yeasts (*Candida* species and *P. kudriavzevii/N. glabrata*) among patients with urogenital infection was significantly higher in females than males (*p* < 0.05). The occurrence of mixed culture of yeasts was observed only in 11 female urogenital samples (8 of the 53 HVS and 3 of the 54 urine samples).

### 3.2. In Vitro Antifungal Susceptibility Testing of Yeast Isolates

The susceptibility of yeast pathogens to antifungal drugs as determined by the agar diffusion method indicated that all the isolates were susceptible to voriconazole and nystatin, while reduced susceptibility was observed for fluconazole (103/129 isolates; 79.84%). The IZDs varied for each drug disk: nystatin (16.0–29.5 mm), voriconazole (17.0–35.5 mm), and fluconazole (0–36.5 mm). *P. kudriavzevii* (*C. krusei*) was the least susceptible species to fluconazole (41.67%) (Table 3). *C. albicans* isolates from males were all susceptible to fluconazole, while reduced susceptibility was observed in yeast isolates from females (93/119 isolates; 78.15%).

The results of MIC for fluconazole are listed in Table 4. Based on the latest published clinical CLSI breakpoints for yeast [33], 17 out of 95 *C. albicans* isolates (~18%) were found resistant to fluconazole followed by *C. tropicalis* with a single isolate showing a MIC value > 64 *μ*g/mL (Table 4). No other yeast isolates were found to be resistant to fluconazole except for *P. kudriavzevii* (*C. krusei*) which is inherently resistant to this drug irrespective of the MIC result (Table 4).

### 3.3. Molecular Identification of *C. albicans* and MLST Genotyping of *hwp1*-Heterozygous Isolates

*hwp1*-PCR identification of green-colored colonies, grown on CHROMagar *Candida*, was done to differentiate *C. albicans* from its phylogenetically closely related yeast: *Candida dubliniensis* and *Candida africana* [24].

**Figure 1:**
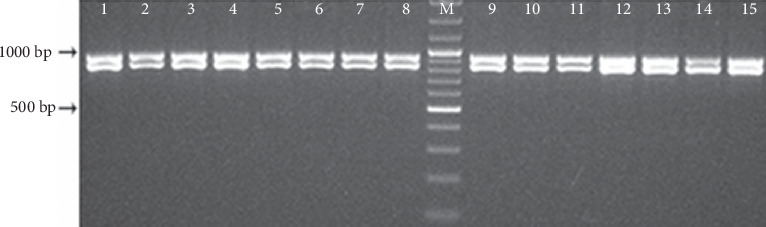
Molecular identification of *C. albicans* isolates heterozygous at *hwp1* locus. Lanes 1–15: PCR products obtained from 15 *hwp1-*heterozygous *C. albicans* isolates recovered in this study (Table 5). Lane M: Molecular size marker, 100 bp.

All isolates were identified as *C. albicans* based on the typical 941-bp amplicon produced by this species [24]. However, it is interesting to note that 15 out of 95 (~16%) Nigerian *C. albicans* isolates yielded an additional DNA fragment of approximately 850 bp indicating that these isolates were heterozygous at *hwp1* locus (Figure 1). MLST genotyping analysis revealed a significant genetic homogeneity among these isolates. In fact, just one allele was found for the following four MLST loci: *ADP1* (Allele 5), *MPIb* (Allele 9), *SYA1* (Allele 2), and *VPS13* (Allele 24) (Table 5), whereas two alleles were detected for the *ZWF1b* locus (Alleles 5 and 20). The *AAT1a* and *ACC1* loci were the most informative with four (Alleles 2, 3, 8, and 31) and three (Alleles 2, 3, and 5) different MLST alleles detected, respectively (Table 5). In total, 13 alleles were identified in this study.

Combining the seven allele numbers for each isolate, we found a total of six different DSTs (Table 5) of which two (DSTs: 52 and 3651) belonged to already known MLST genotypes, while four (DSTs: 4107, 4109, 4114, and 4116) were new and added to the *C. albicans* MLST database. The DST 52 was the most recurrent genotype in our dataset (6/15; 40%), followed by DSTs 4107 (4/15; ~26.7%), 4109 (2/15; 13.3%), and 3651, 4114, and 4116 (1/15; ~6.7% each) (Table 5).

**Figure 2:**
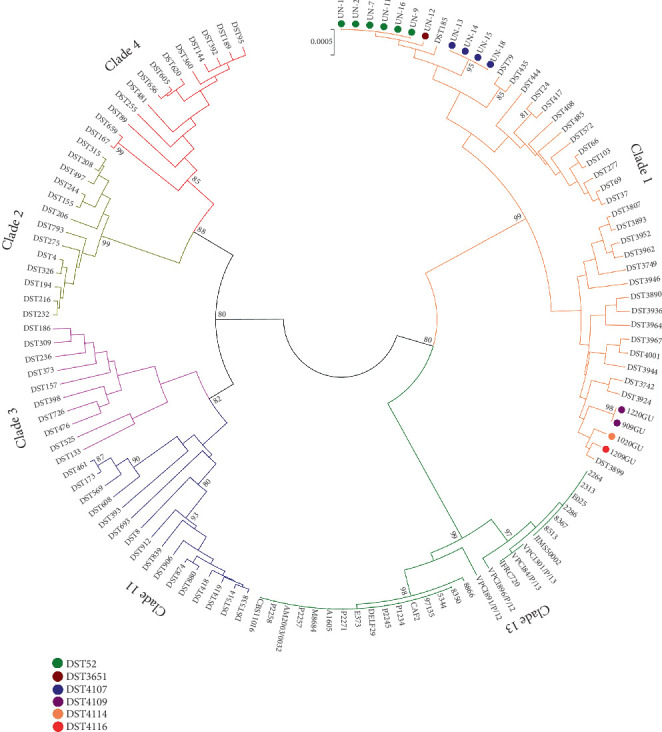
Phylogenetic tree generated by UPGMA analysis using concatenated MLST sequences of Nigerian *C. albicans* isolates and selected DSTs representing the five most populous *C. albicans* clades (1, 2, 3, 4, and 11) and Clade 13. Bootstrap support values above 80% are indicated at the nodes. Nigerian isolates are highlighted by different colors based on their MLST genotype (Table 5).

Phylogenetic analysis, based on UPGMA clustering algorithm, showed that all the DSTs detected in this study clustered close to several isolates previously reported as representatives of the most populous *C. albicans* strain group (Clade 1) [34] (Figure 2).

The goeBURST analysis, conducted at the single locus variant (SLV) level, was performed to compare the allelic profiles of the 15 Nigerian isolates with 3.553 unique DSTs of the 5548 isolates deposited in the *C. albicans* PubMLST database. The minimum spanning tree generated by the PHYLOViZ software grouped the whole dataset into 178 clonal complexes and 1154 singletons. All the Nigerian MLST genotypes clustered into the CC-1, the largest clonal complex found containing 535 DSTs representing 1146 isolates.

## 4. Discussions

In this study, we determined the distribution of *Candida* species, including other pathogenic yeast such as *P. kudriavzevii* (*C. krusei*) and *N. glabrata* (*C. glabrata*), in the urogenital tracts of Nigerian patients with urogenital infection.

Among 248 urogenital samples, a total of 117 samples were positive for yeasts accounting for 47.18%. This result is comparable with data reported by Oladugba who observed a prevalence of 54.55% of candiduria and 63.33% of vaginal infections among females with urogenital infection in Benin City, Nigeria [3]. A prevalence of 48.5% vaginal *Candida* isolates was also reported in Uyo, Nigeria [35]. However, the prevalence observed in the present study was higher than the finding by Adamu et al., who reported a prevalence of 20.8% among women with vulvovaginal infection in Kano, Nigeria [36]. The observed difference might be due to the wide age range (8–52 years) coverage by Adamu et al. which captured nonsexually active patients and patients who tend to menopause or have passed age of childbearing [36]. In the present study, the participants were women of childbearing age, 20–45 years, where *Candida* species are generally the most prevalent [37, 38].

In contrast to the well-known association between *Candida* prevalence and women of childbearing age, Oparaugo et al. reported a low prevalence (12.4%) of VVC and a high bacterial vaginosis (50%) with a coinfection rate of 37.1% among women of childbearing age in Lagos, Nigeria [39]. However, variations in prevalence of *Candida* in urogenital tract also exist in reports from other parts of the world. Venugopal and collaborators reported a prevalence of 34% among patients with genital infection in Saudi Arabia, while Ribeiro et al. observed a prevalence of 60% among patients with symptomatic vaginal infection [40, 41]. The scenario gives evidence that factors such as underlying medical condition, socioeconomic status, ethnicity, sexual activity, and changes in vaginal microflora can influence *Candida* prevalence, supporting the need for proper diagnosis before starting any treatment, especially in cases of urogenital infection [20, 42].

In the present study, the burden of urogenital yeast infection was 54.59% among females (HVS, 56.38; urine 52.94%), while the prevalence in males was 19.23% (US, 25%; urine 20.59%; semen, 0). However, the absence of yeast from the semen samples in this study might be related to the small sample size (six samples). Najjar et al. reported that 28% (17/60) of the semen samples were positive for *Candida* species [43].

Females with urogenital complaint had significantly more *Candida* species isolated (54.59%) than males with urogenital infection (19.23%). No significant difference was noted in the occurrence of *Candida* and *Pichia* in mid-stream urine and genital of patients with urogenital infection (*p* > 0.05). Thus, there was no significant difference between the prevalence of yeasts in the urine and the genitals of humans suffering from urogenital infection.

Mixed cultures of *Candida* species, including *P. kudriavzevii* (*C. krusei*), were found in genitourinary samples. The observation of mixed cultures was possible because the chromogenic culture medium used in this study facilitated the rapid speciation of *Candida* species based on the color assumed by their colonies [44]. A high occurrence (63.64%) of NAC species and *P. kudriavzevii* was observed in mixed culture which were detected in female genitourinary samples only. Males with genitourinary infection did not harbor any mixed cultures of *Candida* species in the genitourinary tract. In our opinion, the higher prevalence of mixed cultures in female samples may be explained by several factors, including the significant bias in the number of isolates collected from females (*n* = 119) compared to males (*n* = 10). Furthermore, as suggested by Jannati et al. [45], urogenital infections caused by multiple *Candida* species may occur due to the development of drug resistance following empiric use of antifungals or may arise secondarily when primary *Candida* infections are not treated promptly.

Among the *Candida* species isolated in this study, *C*. *albicans* (70.15% in urine and 77.42% in genital samples, respectively) was the predominant *Candida* species followed by *C*. *parapsilosis* (7.46% in urine and 4.83% in genital samples) among patients with urogenital *Candida* infection. *P. kudriavzevii* (7.46% in urine and 11.29% in genital samples) occurrence was more than the NAC species. The high prevalence of *C*. *albicans* was previously documented among women with genitourinary complaint in Uyo, Nigeria, by Ikenyi et al. [35], who reported a prevalence of 77.8%, and Oladugba [3] which noted a prevalence of 60% in Benin, Nigeria. However, a shift toward NAC species was reported by Samuel et al., where more than 55% of NAC species were associated with urogenital infection in Lagos, Nigeria [46]. In these studies, variation in the occurrence of NAC spp. was observed [3, 35].

In this study, the antifungal susceptibility testing showed that all fungal isolates were susceptible to nystatin and voriconazole, while 79.84% were susceptible to fluconazole. However, ~18% of *C*. *albicans* isolates exhibited reduced susceptibility to fluconazole (Table 3), an emerging phenomenon already observed and documented by other studies in Africa [3, 10]. Unfortunately, the reasons behind the emergence of azole resistance in African *Candida* isolates are not well understood and could be related to antifungal drug misuse, often promoted by self-medication, unregulated drug sale, *counterfeit*, and adulterated drugs, as well as prescription of antifungal drugs by health practitioners based only on clinical presentation rather than on adequate laboratory diagnosis of the causative agent [10].

In this study, molecular amplification of the *hwp1* gene was used to confirm the presence of *C. albicans* among yeast isolates recovered from urogenital specimens [24]. Among 95 *C. albicans* isolates identified, 15 (~16%) were heterozygous at *hwp1* locus, a singular genotype that has already been previously reported from several countries worldwide [47–51], including Africa [52]. Based on MLST genotyping, our *hwp1*-heterozygous isolates appear to be genetically highly homogeneous and ascribed to six different DSTs, four of which are completely new (Table 5). The remaining two MLST genotypes (DST 52 and 3651) were already present in the official MLST database and recovered out of Africa, one from Austria (DST 3651) and one from United States (DST 52). Interestingly, despite the genetic differences found at the *hwp1* locus, compared to *C. albicans* homozygous strains, *hwp1*-heterozygous strains do not appear to show significant differences in biofilm formation [50] and exoenzyme (phospholipase, hemolysin, and proteinase) production in vitro [51].

In conclusion, to our knowledge, there are no other studies investigating additional virulence factors in these strains, and, although their frequency in clinical samples seems to be *presumably* low, both homozygous and heterozygous strains were responsible for vaginal candidiasis suggesting that further epidemiological investigations, including comparative in vivo and in vitro virulence studies, should be performed to better understand the role of *hwp1*-related differences in vaginal infections.

## 5. Limitations of the Study

The main limitation of this study is the different sample sizes for men and women enrolled. Unequally sized gender groups can lead to unequal variances, a general loss of power and issues with confounding variables making it difficult to generalize the results to the overall population.

## Figures and Tables

**Table 1:** *Candida* species and *N. glabrata* and *P. kudriavzevii* distribution in different urogenital samples.

**Urogenital sample**	**No. sampled**	**Positive samples, ** **n** ** (%)**	**True *Candida* species isolated, ** **n** ** (%)**			***N. glabrata*, ** **n** ** (%)**	***P. kudriavzevii*, ** **n** ** (%)**	**Total**
			***C. albicans***	***C. parapsilosis***	***C. tropicalis***			
Urine	136	61 (44.85)	47 (70.15)	5 (7.46)	5 (7.46)	5 (7.46)	5 (7.46)	67
HVS	94	53 (56.38)	45 (76.27)	3 (5.08)	2 (3.39)	2 (3.39)	7 (11.86)	59
US	12	3 (25)	3 (100)	0	0	0	0	3
Semen	6	0	0	0	0	0	0	0
Total	248	117 (47.18)	95 (73.64)	8 (6.20)	7 (5.43)	7 (5.43)	12 (9.30)	129

*Note:* Yeast occurrence in urine versus genital sample, *χ*^2^ = 20.621; degrees of freedom (df) = 1; *p* > 0.05.

Abbreviations: HVS, high vaginal swab; US, urethral swab.

**Table 2:** Gender and yeast distribution in urogenital infection.

**Source of urogenital sample**	**No. sampled**	**Positive samples, ** **n** ** (%)**	**True *Candida* species isolated, ** **n** ** (%)**			***N. glabrata*, ** **n** ** (%)**	***P. kudriavzevii*, ** **n** ** (%)**	**Total**
			***C. albicans***	***C. parapsilosis***	***C. tropicalis***			
Male	52	10 (19.23)^a^	10 (100)	0	0	0	0	10
Semen	6	0	0	0	0	0	0	0
US	12	3 (25)	3 (100)	0	0	0	0	3
Urine	34	7 (20.59)	7 (100)	0	0	0	0	7
Female	196	107 (54.59)^a^	85 (71.43)	8 (6.72)	7 (5.88)	7 (5.88)	12 (10.08)	119
HVS	94	53 (56.38)	45 (76.27)	3 (5.08)	2 (3.39)	2 (3.39)	7 (11.86)	59
Urine	102	54 (52.94)	40 (66.67)	5 (8.33)	5 (8.33)	5 (8.33)	5 (8.33)	60
Total	248	117 (47.18)	95 (73.64)	8 (6.20)	7 (5.43)	7 (5.43)	12 (9.30)	129

Abbreviations: HVS, high vaginal swab; US, urethral swab.

^a^ *χ* ^2^ = 20.621; degrees of freedom (df) = 1; *p* < 0.05.

**Table 3:** Susceptibility of yeast isolates to antifungal drugs.

**Yeast species (no. of tested isolates)**	**Susceptibility to antifungals, ** **n** ** (%)**		
	**Fluconazole**	**Voriconazole**	**Nystatin**
*C. albicans* (95)	78 (82.11)	95 (100)	95 (100)
*N. glabrata* (*C. glabrata*) (7)	6 (85.71)	7 (100)	7 (100)
*C. parapsilosis* (8)	8 (100)	8 (100)	8 (100)
*C. tropicalis* (7)	6 (85.71)	7 (100)	7 (100)
*P. kudriavzevii* (*C. krusei*) (12)	5 (41.67)	12 (100)	12 (100)
Total	103 (79.84)	129 (100)	129 (100)

**Table 4:** Fluconazole minimum inhibitory concentration with corresponding inhibition zone diameter obtained in this study.

**Yeast species**	**No. of isolates**	**Range of IZD (mm)**	**MIC (*μ*g/mL)**	**CLSI interpretation**
*C*. *albicans*	78	15–36.5	< 8	S
	17	0–14	> 32	R
*N. glabrata* (*C*. *glabrata*)	7	15–23	4–16	S
*C*. *parapsilosis*	8	15–23	< 8	S
*C*. *tropicalis*	6	15–30	2–4	S
	1	0	> 64	R
*P. kudriavzevii* (*C*. *krusei*)	5	15–20	8–16	None^a^
	7	0–12	> 64	None^a^

Abbreviations: CLSI, Clinical Laboratory Standard Institute; IZD, inhibition zone diameter, measured in millimeter; MIC, minimum inhibitory concentration; R, resistant; S, susceptible, which includes susceptible, intermediate, and dose-dependent susceptibility.

^a^All *P*. *kudriavzevii* (*C*. *krusei*) isolates were regarded as fluconazole-resistant irrespective of the MIC result (CLSI, 2022).

**Table 5:** *C. albicans* isolates, allelic profiles, and DSTs generated by MLST analysis.

**Isolate code**	***MLST* loci**							**DST**
	***AAT1a***	***ACC1***	***ADP1***	***MPIb***	***SYA1***	***VPS13***	***ZWF1b***	
**Fragment length** ^**a**^	**373 bp**	**407 bp**	**443 bp**	**375 bp**	**391 bp**	**403 bp**	**491 bp**	
UN-1	2	3	5	9	2	24	5	52
UN-2	2	3	5	9	2	24	5	52
UN-7	2	3	5	9	2	24	5	52
UN-9	2	3	5	9	2	24	5	52
UN-11	2	3	5	9	2	24	5	52
UN-12	31	3	5	9	2	24	5	3651
UN-13	3	3	5	9	2	24	5	4107^b^
UN-14	3	3	5	9	2	24	5	4107^b^
UN-15	3	3	5	9	2	24	5	4107^b^
UN-16	2	3	5	9	2	24	5	52
UN-18	3	3	5	9	2	24	5	4107^b^
909GU	2	5	5	9	2	24	20	4109^b^
1020GU	8	5	5	9	2	24	20	4114^b^
1209GU	8	2	5	9	2	24	20	4116^b^
1220GU	2	5	5	9	2	24	20	4109^b^

Abbreviation: DST, diploid sequence type.

^a^Length of nucleotide sequences obtained by Sanger sequencing.

^b^New DST.

## Data Availability

The data that support the findings of this study are openly available in *Candida albicans* PubMLST database at https://pubmlst.org/organisms/candida-albicans. Moreover, the data that support the findings of this study are available from the corresponding authors upon reasonable request.

## Supporting Information

Supporting Information 1 Table S1: Primers and PCR conditions used for molecular identification of fungal species retrieved in this study.

Supporting Information 2 Figure S1: Electrophoresis gel showing the molecular identification of *N. glabrata.*
