# Supplementary material for: Trends in Prevalence of Yeast Species Associated With Urogenital Infection in Nsukka, Nigeria: An Overview of True Candida Species and Genotyping of Candida albicans hwp1-Heterozygous Isolates
Source: Int J Microbiol. 2025 Sep 17;2025:3115363. doi: 10.1155/ijm/3115363 (PMC12460009; doi:10.1155/ijm/3115363)
Supplement: Supporting Information 1 — Table S1: Primers and PCR conditions used for molecular identification of fungal species retrieved in this study. [file 3115363.f1.docx]

| Table S1: Primers and PCR conditions used for molecular identification of fungal species retrieved in this study. | | | | |
| --- | --- | --- | --- | --- |
| Primers Sequence | PCR Conditions | Target | Species-specific amplicon size | Reference |
| CR-f: 5′-GCTACCACTTCAGAATCATCATC-3′  CR-r: 5′ GCACCTTCAGTCGTAGAGACG-3′ | Initial denaturation at 95 °C for 5 min  30 cycles of  Denaturation at 94°C for 45s  Annealing at 58 °C for 40 s  Extension at 72 °C for 55 s  Final Extension step at 72 °C for 10 min | *HWP1* | *C. albicans*: *~*1000 bp  *C. africana*: *~*700 bp  *C.dubliniensis*: *~*500 bp | [24] |
| VMA‐f: 5’-AATCCGAAGGCTTGATGG-3’  VMA‐r: 5’-AATGCCAGCAGCAAAAGTAG-3’ | Initial denaturation at 95°C for 5 min  30 cycles of  Denaturation at 94°C for 1 min  Annealing at 53°C for 40 sec  Extension at 72°C for 45 sec  Final Extension step at 72°C for 7 min. | VMA | *C. tropicalis*: *~*245 bp | [25] |
| GLA-f: 5’-CGGTTGGTGGGTGTTCTGC-3’  NIV-f: 5’-AGGGAGGAGTTTGTATCTTTCAAC-3’  BRA-f: 5’-GGGACGGTAAGTCTCCCG-3’  UNI-5.8S-r: 5’-ACCAGAGGGCGCAATGTG-3’ | Initial denaturation at 95 °C for 5 min 34 cycles of  Denaturation at 94 °C for 30s  Annealing at 60 °C for 40s  Extension at 72 °C for 50s  Final Extension step at 72 °C for 10 min. | ITS1  ITS1  ITS1  5,8S | *N. glabrata: ~400* bp  *C. bracarensis*: *~230* bp  *C. nivariensis: ~300* bp | [26] |
| mCP-F: 5’-TTTGCTTTGGTAGGCCTTCTA-3’  mCO-F: 5’-TAAGTCAACTGATTAACTAAT-3’  mCM-F: 5’-AACTGCAATCCTTTTCTTTCTA-3’  mCPC-R: 5’-AATATCTGCAATTCATATTACT-3’ | Initial denaturation at 95°C for 5 min  30 cycles of  Denaturation at 95°C for 1 min  Annealing at 52°C for 30 s  Extension at 72°C for 1 min  Final Extension step at 72°C for 10 min. | ITS1  ITS1  ITS1  5,8S | *C. parapsilosis: ~170* bp  *C. orthopsilosis: ~110* bp  *C. metapsilosis: ~220* bp | [27] |
