# Supplementary material for: Trends in Prevalence of Yeast Species Associated With Urogenital Infection in Nsukka, Nigeria: An Overview of True Candida Species and Genotyping of Candida albicans hwp1-Heterozygous Isolates
Source: Int J Microbiol. 2025 Sep 17;2025:3115363. doi: 10.1155/ijm/3115363 (PMC12460009; doi:10.1155/ijm/3115363)
Supplement: Supporting Information 2 — Figure S1: Electrophoresis gel showing the molecular identification of N. glabrata. [file 3115363.f2.docx]

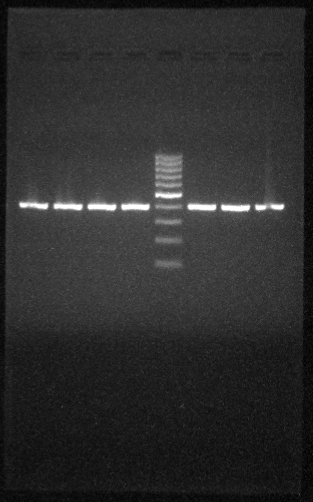


**Supplementary Figure 1**: Electrophoresis gel showing the molecular identification of *N. glabrata.* All the isolates retrieved in this study produced an amplicon of ~400bp according to the mPCR protocol described by Romeo et al. [26]. Molecular size marker, 100-bp.
